# 

**Published:** 1819-05

**Authors:** 


### MONTHLY CATALOGUE OF MEDICAL BOOKS.

An Arrangement of British Plants, according to the latest Improvements of the Linnæan System; with an easy Introduction to the Study of Botany. Corrected and considerably enlarged, by Wm. Withering, Esq. F.L.S. 4 vols. 2l. 8s.

John Gottlieb Walter's Plates of the Thoracic and Abdominal Nerves, reduced from the original, as published by order of the Royal Academy of Sciences at Berlin. 4to. 15s.

Lectures on Physiology, Zoology, and the Natural History of Man. By Wm. Laurence, F.R.S. Professor of Anatomy and Surgery to the College, &c. 8vo. 1l. 1s.

Laurentii Io. Rubi Epistolarum Edinburgensium, Libri III. Written during three years' attendance on the Medical Institution of that City. 12mo. 5s.

Medical Topography of Upper Canada. By John Douglas, assistant-Surgeon, 8th Regiment. 8vo. boards, 4s. 6d.

Observations on the History and Treatment of the late Epidemic Disorder which prevailed in the North of Ireland. By F. Rogan, M.D. 6s.

A Manual of Chemistry. By W. T. Brande. 8vo. 25s.

Cases, with Observations on Wry-Neck; on the Reduction of Luxation of the Shoulder-Joint; on the Operation for Hare-Lip; on Cartilaginous Substances in the Knee-Joint; on Aneurism, &c. By John Kirby, A.B. Member and one of the Censors of the Royal College of Surgeons in Ireland, &c. 6s.

A Narrative of a Voyage to New South Wales, in the year 1816, in the ship Mariner, describing the Nature of the Accommodations, Stores, Diet, &c.; together with an Account of the Medical Treatment, &c. By John Haslam, jun. Surgeon, Royal Navy. 8vo. 1s.

A Letter to the Rev. Thomas Rennell, concerning his Remarks on Scepticism; from a Graduate in Medicine, of the University of Oxford. 8vo. 2s. 6d.

Medical Botany; or, the History of the Plants in the Materia Medica, No. IV. Royal 8vo. 3s. 6d.

